# Modelling Shows the Negative Impact of Age Dependent Pharmacokinetics on the Efficacy of Oxytetracycline in Young Steers

**DOI:** 10.3389/fvets.2021.821005

**Published:** 2022-01-28

**Authors:** Peter Hekman, Johan Schefferlie, Ronette Gehring

**Affiliations:** ^1^Medicines Evaluation Board, Veterinary Medicines Unit, The Hague, Netherlands; ^2^Veterinary Pharmacotherapy and Pharmacy, Department of Population Health Sciences, Utrecht University, Utrecht, Netherlands

**Keywords:** PBPK, PK/PD, oxytetracycline, bovine, clearance, efficacy

## Abstract

The effect of age dependent pharmacokinetics (PK) on the clinical efficacy of oxytetracycline (OTC) against Bovine Respiratory Disease (BRD) in beef cattle was studied, using a Physiologically Based Pharmacokinetic (PBPK) model. The model includes a bodyweight dependent renal clearance. To mimic/reproduce the long terminal half-live a bone forming tissue compartment was considered. Data for the development, calibration and validation of the model were obtained from public literature. To integrate the PK with the pharmacodynamics (PD) of OTC, Monte Carlo simulations were performed using this PBPK model to predict time-concentration curves for two commonly used dosing regimens of short-acting and long-acting injectable OTC formulations in virtual populations of 5,000 steer calves of 100 kg and 400 kg. These curves were then used to calculate the value of the PKPD index for OTC, which is the ratio of the area under the concentration-time curve for 24 h (AUC_24h_) over the minimum inhibitory concentration (MIC) of the target pathogen (AUC_24h_/MIC). The MIC values were for *Mannheimia haemolytica*, the dose-limiting pathogen for BRD. This integration of PBPK and PD for OTC used for the treatment of BRD in calves indicated that the Probability of Target Attainment (PTA) was sufficient for efficacy in calves of 400 kg, but insufficient for calves of 100 kg, when using a long acting dosing regimen of 20 mg/kg BW, twice, with a 48-h interval. The use of a dosing regimen of 10 mg/kg BW/day for 4 days predicted sufficient PTAs in both age groups.

## Introduction

Dose determination for veterinary medicinal products (VMPs) is commonly performed under controlled experimental conditions in an often homogeneous subset of the target population. Such studies do not normally address the effects of important factors that can influence the pharmacokinetics (PK), such as age, production stage, health status, or breed. It would be impractical to study the effects of these factors individually and in combination for all VMPs. Instead, clinical field studies are performed that presumably cover a representation of the total target population, and the clinical benefit for the total study population is statistically evaluated against negative and/or positive controls. However, this approach could still neglect the PK of specific subpopulations, and does not determine whether or not adjustments to the dosage regimen is required for subpopulations to achieve concentrations that maximise the probability of a positive clinical outcome.

The influence of factors like age, production stage or breed on the PK of VMPs can be clinically relevant. For example, Mzyk et al. (1) showed marked differences in the area under the plasma concentration-time curve (AUC) and clearance of subcutaneously (s.c.) administered danofloxacin or tulathromycin in 3-weeks old vs. 6-month old calves. Igarza et al. (2) investigated the PK of intravenously (i.v.) administered ketoprofen in cows in early lactation, pregnant cows, and newborn calves, and found significant differences between these groups in elimination half-life, mean residence time, and AUC. Gorden et al. (3) showed that meloxicam persisted at higher concentrations in the plasma for longer in post-partum vs. mid-lactation dairy cows. This difference was shown to be caused by a 2-fold lower hepatic clearance in post-partum cows (4) and may, according to the authors, necessitate the adjustment of dose and/or withdrawal period (WP). Chang et al. (5), reported differences in plasma concentrations of orally administered florfenicol in Leghorn chickens vs. Taiwan native chickens. They also determined the residue depletion in tissues and found that florfenicol residues persisted longer in Taiwan native chickens. The authors suggested that the WP for florfenicol in Leghorn chickens may not be safe in Taiwan native chickens. Howard et al. (6) studied different pig breeds and found significant differences in clearance and volume of distribution for i.v. administered flunixin meglumine, and in AUC for i.v. administered oxfendazole, indicating differences in hepatic drug clearance.

Physiologically Based Pharmacokinetic (PBPK) modelling is an alternative approach to exploring the PK of VMPs to discover how dosage regimens need to be adjusted to account for the influence of various factors. In such models, parameters such as (age-dependent) body composition and hepatic and renal clearance can be varied to determine the effects on the PK. This approach can address the variation within the target population without the need for *in vivo* studies.

We constructed a PBPK model for oxytetracycline (OTC) in beef cattle. OTC was chosen because it has very limited hepatic clearance and is excreted in urine (7). Because the glomerular filtration rate (GFR) in cattle changes with age, OTC is likely to have an age dependent clearance. Using this PBPK model, validated with data from public literature, the plasma concentration-time profiles of OTC were predicted for the two common dosage regimens of short and long acting formulations in steers of different ages. In order to determine if the age-dependent differences in PK could have an impact on the efficacy, a PK/PD analysis was performed.

## Materials and Methods

### Data Sources

#### General

The data for the construction, calibration and validation of the PBPK model were obtained from literature. Plasma concentration-time data of published studies were (when needed) extracted from graphs of the selected studies using a digital tool developed at the Medicines Evaluation Board, Veterinary Medicinal Products Unit, the Netherlands, that converts graphical data into digital time-concentration data.

#### Data Used for Model Development

Most of the data originate from published PK studies in steer calves of various ages and bodyweights. Two published studies in dairy cows were used for estimating the urinary excretion rate. Table 1 provides an overview of the data used.

**Table 1:** Listing of the studies and the way that data from these studies were used during the development and validation of the model.

**Study references**		**Mean BW (kg)**	n^*^	**Dose (mg/kg)**	**Product Conc. OTC (mg/ml)**	**Route**	**Purpose**
De Laistre Banting (8)	steer	58	14	10	50	s.c.	I
Meyer et al. (9)	steer	106	5	40	100	i.v.	I, III
Meyer et al. (9)	steer	106	5	20	100	i.m.	I, II, III
Nouws et al. (7)	cow	578	16	5	100	i.v./i.m.	I
Nouws and Vree (10)	steer	n.s.^**^	6	18	100	s.c./i.m.	II, IV
Mevius et al. (11)	cow	570	8	11	200	i.m.	I
Terhune and Upson (12)	steer	300	5	40	200	i.m.	I, II
Achenbach (13)	steer	396	6	20	200	i.m.	I, II, III
Achenbach (13)	steer	396	6	20	300	i.m.	I, II, III
Clarke et al. (14)	steer	336	6	20	200	i.m.	I, II
Toutain and Raynaud (15)	steer	243	6	20	200	i.m.	II, IV
Lees et al. (16)	steer	179	10	20	200	i.m.	IV
Breeze and Gay (17)	steer	308	4	20	200	i.m.	IV
Breeze and Gay (17)	steer	340	4	2 x 20	200	i.m.	IV
Breeze and Gay (17)	steer	342	4	40	200	i.m.	IV
Davey et al. (18)	steer	200	5	20	200/100	i.m.	IV
Craigmill et al. (19)	steer	242	4	20	200	i.m.	IV

* *n, number of animals*.

** *n.s., not specified (age 14 weeks; 100–120 kg BW was used)*.

*I: data used for modelling age/body weight dependent renal clearance of OTC*.

*II: data used for the estimation of the absorption rate*.

*III: data used for the calibration/fitting of the model*.

*IV: data used for the validation*.

General age dependent data on physiology in calves were taken from Lin et al. (20), Lautz (21), and for the GFR in bovine from Murayama et al. (22).

### Software

The PBPK simulations were made using a fit for purpose version of Multisim, a computer program developed at the Medicines Evaluation Board, Veterinary Medicinal Products Unit, the Netherlands. The program is based on the algorithms of Yamaoka and Nakagawa (23), capable of solving sets of predefined differential equations in which time acts as an independent variable. The program uses Gill's fourth order method, a Runge-Kutta method for approximating the solution of the initial value problem y'(x) = f(x,y); y(x0) = y0 which evaluates the integrand f(x,y) 4 times per step.

For the purpose of this paper, the program was equipped with the possibility of performing Monte Carlo simulations.

### Model Construction

**Figure 1:**
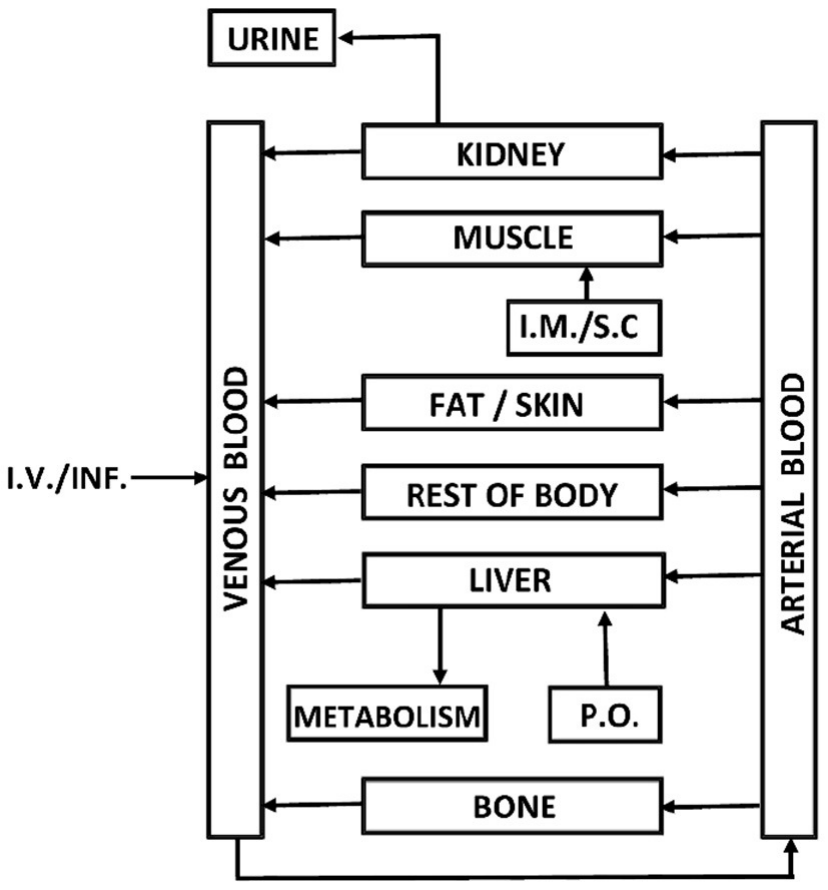
Schematic representation of the PBPK model.

Figure 1 shows a schematic representation of the flow-limited PBPK model. The model is based on the generic models published in literature (21, 24).

Some adjustments to the generic model were made in order to make it fit for purpose:

1. The tissue compartments that were not of interest were lumped together in a compartment called “rest of body,” similar to the model described by Li et al. (24).
2. To appropriately represent the physiology of the kidney function, the renal excretion rate was made dependent on the renal arterial blood concentration rather than the concentration in the kidney compartment.
3. Instead of clearance constants (L/h), the absorption processes after oral (p.o.) or intramuscular/subcutaneous (i.m./s.c.) administration were described in terms of rate constants (1/h). This allows relating the absorption rate to the amount of drug at the site of administration and not to its concentration.
4. In order to successfully describe the prolonged terminal phase in the plasma depletion pattern, a bone forming tissue compartment (“bone” in the model) was introduced. Although it was noted that no accumulation of OTC was found in bone marrow (25), relatively high OTC residues in bone were reported in literature [e.g. (26–28). These data seem to confirm OTC's potential to accumulate in bone forming tissue, as well as its (slow) elimination over time.

These adjustments lead to the following set of differential equations, describing the model:


(1) $$\begin{array}{l} \text{dMi}/\text{dt}=\;\text{COtot}\;.\;\text{fCOi}\;.\;(\text{C}\text{art}-\text{Ci}/\text{Pi})\\ \left(\text{for}\;\text{compartments}\;\text{fat},\;\text{rest}\;\text{body}\;\text{and}\;\text{bone}\right) \end{array}$$

(2) $$\begin{array}{l} \text{dMkidney}/\text{dt}=\;\text{COtot}\;.\;\text{fCO}\text{kidney}\\ .\;(\text{Cart}-\text{C}\text{kidney}/\text{P}\text{kidney})-\text{Kel}1.\;\text{Cart} \end{array}$$

(3) $$\begin{array}{l} \text{dMmuscle}/\text{dt}=\;\text{COtot}\;.\;\text{fCOmuscle}\\ .\;(\text{C}\text{art}-\text{C}\text{muscle}/\text{P}\text{muscle})+\;\text{Rabs}(\text{im}/\text{sc}).\;\text{M}(\text{im}/\text{sc}) \end{array}$$

(4) $$\begin{array}{l} \text{dMliver}/\text{dt}=\;\text{COtot}\;.\;\text{fCO}\text{liver}\;(\text{Cart}-\text{C}\text{liver}/\text{P}\text{liver})\\ +\;\text{Rabs}(\text{po}).\;\text{M}(\text{po})\;-\text{Kel}2\;.\;\text{Cliver} \end{array}$$

(5) $$\begin{array}{l} \text{dMvenous}\;\text{blood}/\text{dt}=\;\sum \text{i}\;(\text{COtot}\;.\;\text{fCOi}\;.\;\text{Ci})\\ -\;\text{COtot}\;.\;\text{C}\text{ven} \end{array}$$

(6) $$\begin{array}{l} \text{dMarterial}\;\text{blood}/\text{dt}=\;\text{COtot}\;.\;\text{Cven}\\ -\;\text{COtot}\;.\;\text{Cart} \end{array}$$

(7) dM(im/sc)/dt= −Rabs(im/sc). M(im/sc)

(8) dM(po)/dt= −Rabs(po). M(po)

Where:

- C_art_: concentration in arterial blood (mg/L)
- C_i_: concentration in compartment i (mg/kg)
- fCOi: tissue blood flow (L/h.kg)
- CO_tot_: cardiac output (L/h.kg)
- C_ven_: concentration in venous blood (mg/L)
- M_i_: amount in compartment i (mg)^1^
- P_i_: tissue/blood partition coefficient for compartment i
- R_abs_: absorption rate constant (1/h)
- t: time (h)
- Kel_1_: renal clearance (L/h.kg)
- Kel_2_: hepatic clearance (L/h.kg).

### Model Parameters

#### General

The values of the physiological parameters (tissue blood flows, relative tissue weights and volumes) and their co-variance were directly obtained or calculated from review articles (13, 20, 21). The values of these parameters were fixed and are summarised in Table 2. It should be noted that these parameters are only valid for commonly used breeds of cattle (e.g. Friesian, Jersey, Angus, Hereford, and Holstein).

**Table 2:** Parameter values for calves 100–200 kg and steers > 200 kg derived from literature.

**Tissue**	**Partition**	fCO^*^	fVol^*^
blood			0.07 (0.038)
kidney	7	0.1	0.004 (0.002)
Liver:	3.5	0.3 (0.44)	0.003 (0.012)
Fat:	0.12	0.068	0.07 (0.184)
Muscle:	0.56	0.28	0.34 (0.36)
${\text{Kel}}_{1}=220\text{ x }0.0054\text{ x B}{\text{W}}^{\wedge }-0.47\text{ L}/\text{h}.\text{kg};\text{COtot}=9.1\text{L}/\text{h}.\text{kg};$			
(*COtot* = 5.45*L*/*h*.*kg*)			

* *In parenthesis parameters for steers. >200 kg (when different)*.

#### Partition Coefficients

The partition coefficients were calculated as the ratio Ctissue/Cplasma for muscle, kidney, liver and fat from data in literature (13) at day 4 post administration of a s.c. dose of 20 mg/kg OTC to 400 kg steer calves.

For the partition coefficient for bone, the following approach was taken: (9) calculated that the final t½ in calves was approximately 95 h after either i.m. or i.v. administration. In the current model, this terminal phase in the plasma curve is assumed to be caused by the elimination rate of OTC from the bone compartment after it had been accumulated there. The elimination half-life from this compartment can be expressed as:


(9) 1.44×t½=fVbone /( fCObone .COtot/Pbone)

Therefore:


(10) Pbone=1.44 . t½ . (fCObone.COtot/fVbone)

#### Relative Organ Weights and Blood Flows Not Found in Literature

The starting value for fVol of the bone forming compartment used in the fitting process was assumed to be 1% of the bodyweight, being ~10% of the mean value reported for total bone in literature (20, 21).

Since no data were found for cattle, the starting value for fCO of the bone forming compartment used for fitting was assumed to be 1% of the total cardiac output/bodyweight. This is approximately 10% of the value reported in man for total bone (29).

#### Renal Clearance (Kel_1_)

The overall renal clearance of OTC in cows was found to be related to the glomerular filtration rate (GFR). It was concluded that other processes, like passive diffusion and active secretion, could be involved too (7).

Murayama et al. (22) measured the GFR in Holstein cattle of different age/ bodyweights (BW). They expressed the GFR as ml/min.BSA (BSA = body surface area).They measured a mean GFR of 13.2 L/h.m^2^. In order to express this as L/min/h.kg BW, the relation between BSA and BW was used (Equation 11).

Since the renal clearance of OTC is mainly related to the GFR and other processes (e.g. active transport and passive diffusion) may also play a role, we introduced a factor A in order to be able to fit the data on renal clearance found in literature according to Equation 12.


(11) $$\text{BSA}\;=0.09\times {\left[\text{BW}\right]}^{\wedge }(0.667)({\text{m}}^{2})$$

So Kel_1_ could be calculated as:


(12) $$\text{Kel}1\;=13.2\times 0.09\times {\left[\text{BW}\right]}^{\wedge }(0.667\text{A})/\text{BW}\;(\text{L}/\text{h}.\text{kg})$$

Table 3 shows the references and input data for fitting.

**Table 3:** References and input data for fitting the renal clearance.

**References**	**Mean BW (kg)**	**CL renal (L/h.kg)**
De Laistre Banting (8)^*^	58	0.16
Meyer et al. (9)^*^	106	0.135
Meyer et al. (9)^*^	106	0.131
Terhune and Upson (12)^*^	300	0.087
Clarke et al. (14)^*^	340	0.081
Achenbach (13) (1)^*^	396	0.078
Achenbach (13) (2)^*^	396	0.083
Mevius et al. (11)^**^	570	0.058
Nouws et al. (7)^**^	580	0.076

* *Assuming for OTC, that renal excretion is the only route of elimination and further a bioavailability of 100%, the renal clearance was calculated from plasma data as: CLrenal = Dose (mg/kg)/AUC (mg.h/L)*.

** *Measured in cows as: urinary excretion rate (mg/h.kg)/plasma concentration (mg/L)*.

**Figure 2:**
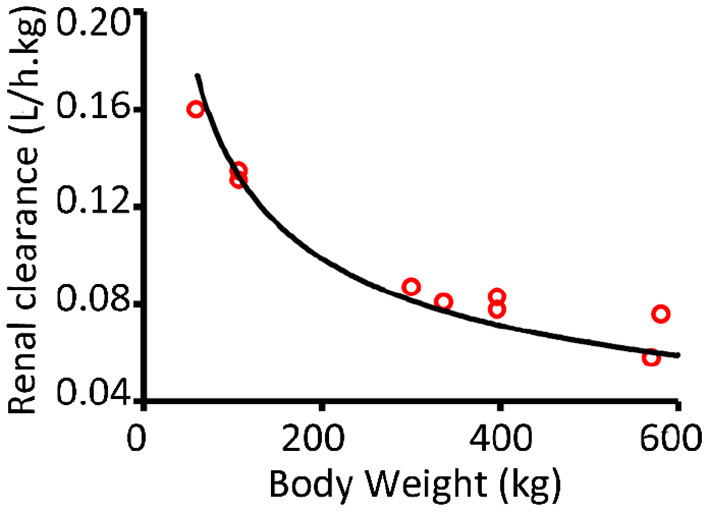
Renal clearance curve fitted according to Equation 12.

After fitting the data to Equation 12 (see Figure 2), Factor A was estimated to be 0.795.

#### Parameters Left for Estimation/Refinement

OTC shows very little biotransformation, with up to 90% of a dose being excreted in urine during the first 72 h after dosing (7). Therefore, Kel_2_ was assumed to be zero.

The initial parameter values for the model are given in Table 2.

The variables left to be estimated or refined through fitting were:

- P(rest-of-body); [NB fCO_6_ and fVol_6_ are fixed parameters and can be calculated respectively as: (1-∑fCO_i_; 1-∑fVol_i_)].
- R_abs_
- fraction of volume (fVol_bone_)
- fraction blood flow (fCO_bone_)
- (P_bone_)

These parameters were estimated via fitting the experimental data from Achenbach (13) and Meijer et al. (9) who followed the time dependent plasma concentrations of OTC after i.v., i.m. and s.c. administration to groups of steer calves (Meyer data 106 kg BW, Achenbach data 396 kg BW). Unfortunately the data from Achenbach for the s.c. administrations could not be used, because of the missing bodyweight values.

### Results of Calibration

Table 4 shows the results of the parameters that were fitted.

**Table 4:** Final values of the parameters for 100 kg BW and 400 kg BW steers.

40 mg/kg i.v. and 20 mg/kg i.m. 100 kg BW steers [using data from (9)]			
**Tissue**	**Partition**	**fCO**	**fVol**
Bone forming tissue	400	0.002 (i.v.) 0.003 (i.m.)	0.01
Rest of body	3.3		
Kel_1_ = 0.13 L/h.kg; R_abs_ = 0.24 h^−1^			
20 mg/kg 400 kg BW steers [using 2 sets of data from (13)]			
**Tissue**	**Partition**	**fCO**	**fVol**
Bone forming tissue	400	0.008	0.01
Rest of body	4		
Kel_1_ = 0.072 L/h.kg; R_abs_ = 0.16 h^−1^			

**Figure 3:**
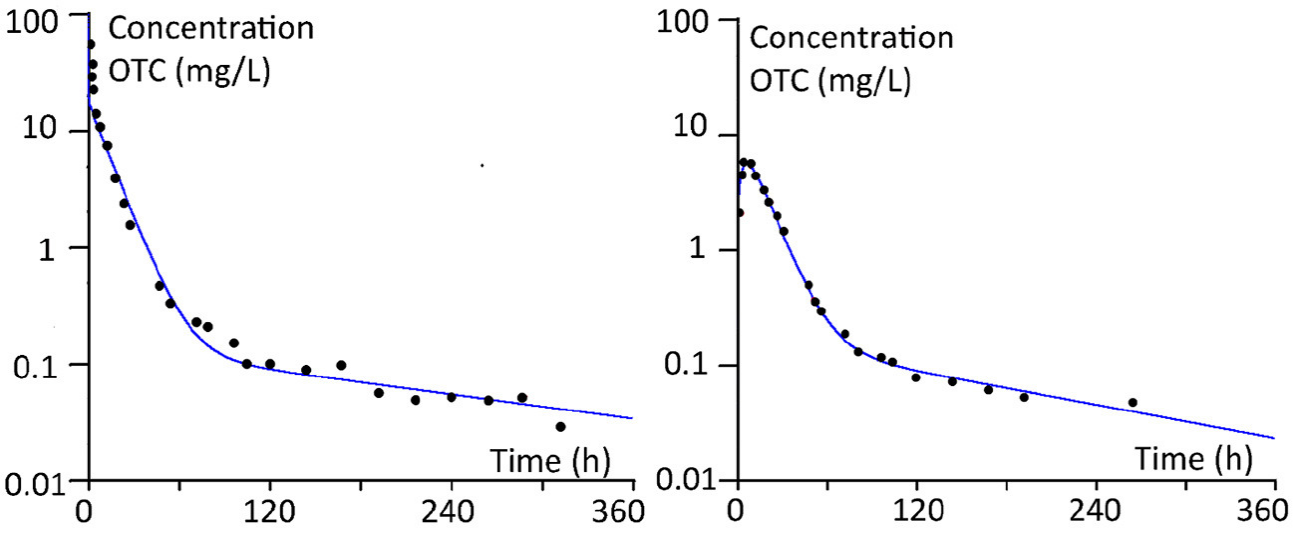
Plasma concentrations and the curves fitted according to the model parameters for calves (100 kg BW), after i.v. and i.m. administration.

As an example, Figure 3 shows the plasma concentrations and the curves fitted according to the model parameters for calves (100 kg), after i.v. and i.m. administration. It should be noted that the PBPK model used is a flow-limited model. Therefore the initial distribution phase after i.v. administration could not be covered as adequately as with a permeability-limited model.

Since the absorption rate from injection sites is known to show a relatively large variability, we determined this R_abs_ for a number of other datasets too. For simulation purposes we used the final values of the already fitted parameters, leaving the absorption rate as the only parameter to fit. This way we could get an indication of the mean and covariance for this parameter. Table 5 shows the results.

**Table 5:** Overview of the results and data used for estimation of R_abs_.

**References**	**Route adm**	**Conc OTC in Product (mg/ml)**	**Dose (Mg/kg)**	**Inj vol/inj site (ml)**	**Number injection sites**	**R_**abs**_ (1/h)**
Achenbach (13)	i.m.	200	20	15 ml	3	0.16
Terhune and Upson (12)	i.m.	200	40	10 ml	5	0.14
Achenbach (13)	i.m.	300	20	10 ml	3	0.16
Toutain and Raynaud (15)	i.m.	200	20	12 ml	2	0.12
Meyer et al. (9)	i.m.	100	20	n.a.	n.a.	0.24
Nouws and Vree (10)	i.m.	100	18	18 ml	1	0.17

The mean absorption rate was 0.17 ± 0.04 h^−1^.

#### Final Sets of Parameters After Calibration

Table 6 shows an overview of the parameters resulting from the calibration process.

**Table 6:** Final sets of parameter values [mean (CV%)] for 100–200 kg BW and >200 kg BW steers from literature or after fitting.

Steer Calves 100–200 kg BW; COtot = 9.1 L/h.kg (30)			
**Tissue**	**Partition**	**fCO**	**fVol**
blood	1	1	0.07 (5)
kidney	7 (20)^*^	0.1 (30)	0.004 (18)
Liver	3.5 (20)^*^	0.3 (37)	0.003 (12)
Fat	0.12 (20)^*^	0.068 (24)	0.07 (28)
Muscle	0.56 (20)^*^	0.28 (32)	0.34 (5)
Bone forming tissue	400^**^ (20)^*^	0.003^**^ (20)	0.01 (20)
Rest of body	3.3^**^ (20)^*^	0.249 (20)	0.503 (20)
Kel_1_ = 220 x 0.0054 x BW^−^0.47 L/h.kg (20)^*^			
R_abs_ _overall_ = 0.17^**^ (30)^*^			
Steer Calves >200 kg BW; COtot= 5.45 L/h.kg (27)			
**Tissue**	**Partition**	**fCO**	**fVol**
blood	1	1	0.038 (17)
kidney	7 (20)^*^	0.1 (72)	0.002 (24)
Liver	3.5 (20)^*^	0.44 (57)	0.012 (15)
Fat	0.12 (20)^*^	0.068 (24)	0.184 (40)
Muscle	0.56 (20)^*^	0.28 (32)	0.36 (32)
Bone forming tissue	400^**^ (20)^*^	0.008^**^ (20)	0.01 (20)
Rest of body	4^**^ (20)^*^	0.104 (20)	0.394 (20)
Kel_1_ = 220 x 0.0054 x BW^−^0.47 L/h.kg (20)^*^			
R_abs_ _overall_ = 0.17^**^ (30)^*^			

*Figures in parenthesis are % coefficient of variation*.

* *Lognormal distribution*.

** *Model fitted parameter; estimated by fitting the PBPK model with the pharmacokinetic data. These parameters were marked as “model fitted” (9, 13)*.

### Model Validation

**Figure 4:**
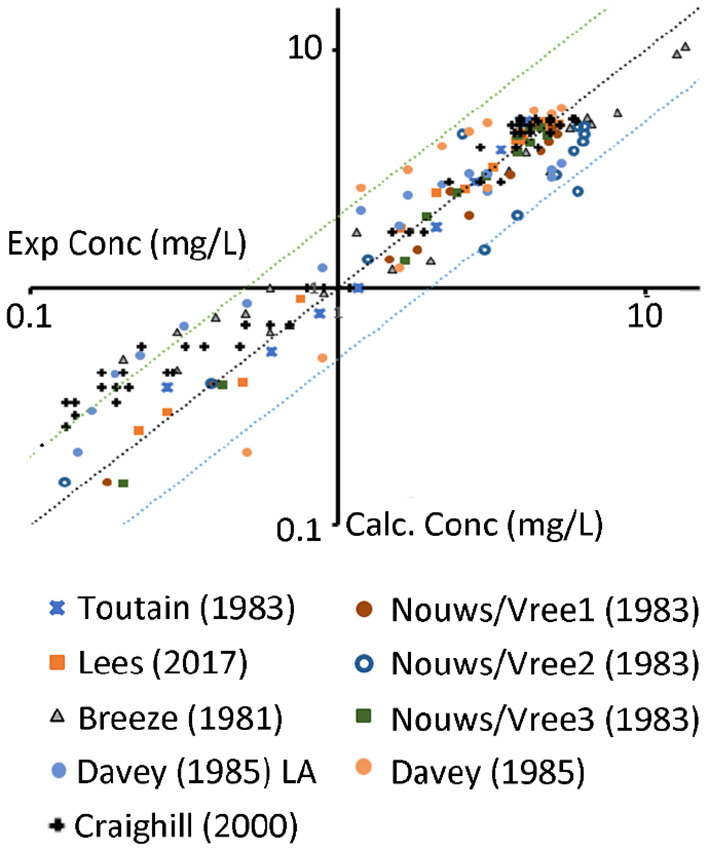
Experimental data (x-axis) vs. predictions (y-axis) of the data from the references above. The middle line represents unity; the lines above/below represent the 2 fold changes. Nouws/Vree 1,2,3 represent different sites of administration (1 neck, 2 shoulder, 3 buttock). Davey represents data from a Long acting (LA) 100 mg/ml product.

In order to get a sense of its predictive power, using the final sets of parameters, the calibrated model was used to simulate various other plasma/serum data obtained from public literature (see Figure 4 and references in Table 1).

### PBPK Model Application

The PBPK model, with its final set of parameters, was used to simulate the time dependent course of the plasma concentrations of OTC for two different commonly used dosing regimens in steer calves for the treatment of respiratory disease (BRD). These two dosing regimens are:

- dosing 10 mg/kg once a day for 4 days via i.m. injection (“short-acting”).
- dosing 20 mg/kg twice with an interval of 2 days via i.m. injection (“long-acting”).

Using Monte Carlo simulations, during a 4 dosing day period, for each dosing regimen, the median plasma curve and the daily AUC_24h_ for the 12 weeks old (100 kg) steer calves were compared to those of the 54 weeks old (400 kg) ones. The distribution of the AUCs obtained from the MC simulation was found to be normally -distributed based on the Q-Q plots. Therefore the standard deviation of the median AUCs could be determined as AUC 50th percentile—AUC 84.1th percentile.

### PK/PD Analysis

A PK/PD analysis was performed to study the influence of the age-dependent PK of OTC on the efficacy of both commonly used dosing regimens in steers of different ages, in case OTC was used for the treatment of Bovine Respiratory Disease (BRD). It was considered that *Mannheimia haemolytica* was the dose limiting pathogen for BRD and that the relevant pharmacodynamic index for OTC was AUC_24h_/MIC, with target values of 42 h and 59 h for the bacteriostatic effect and the bactericidal effect, respectively (30).

Monte Carlo simulations were performed (5,000 runs each), in which the variation of the AUC_24h_ and AUC_24h_/MIC for each dosing regimen was derived from runs of the PBPK model with random sampling from the distributions of the model parameters (see Table 6), and in which the variation in the MIC was based on random sampling from the frequency distribution for MICs of the wildtype strains as reported in the scientific literature (31, 32). To ensure that the randomly selected parameters of the PBPK model are biologically plausible, in the simulations the 2.5th and 97.5th percentiles of each parameter distribution were used as the upper and lower bounds for sampling. The results were used to calculate the probability of target attainment (PTA) for the bacteriostatic and bactericidal effects (as a percentage of the target values indicated above).

## Results

### Modelled PK of Commonly Used Dosing Regimens for OTC

**Figure 5:**
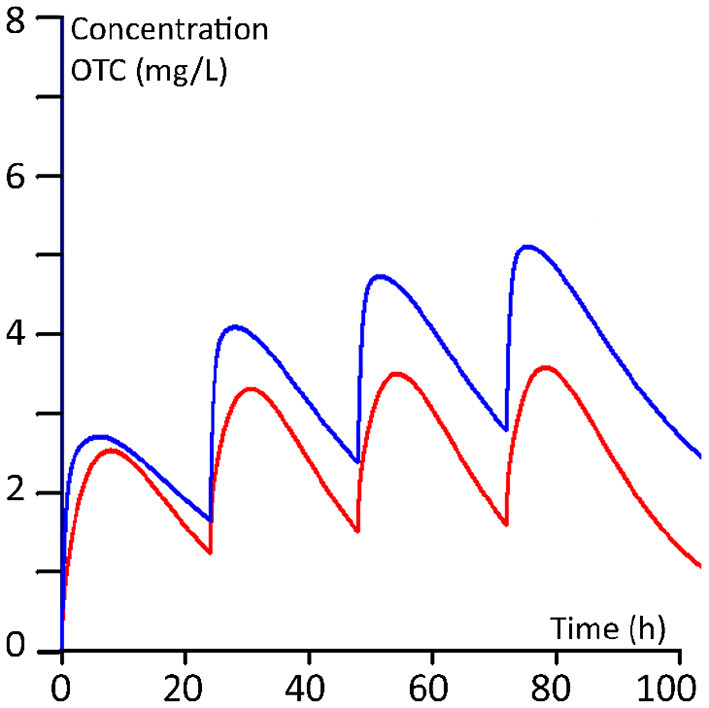
Ten mg/kg regimen (once daily for 4 days). Red line: 100 kg BW calves; blue line 400 kg BW calves.

**Figure 6:**
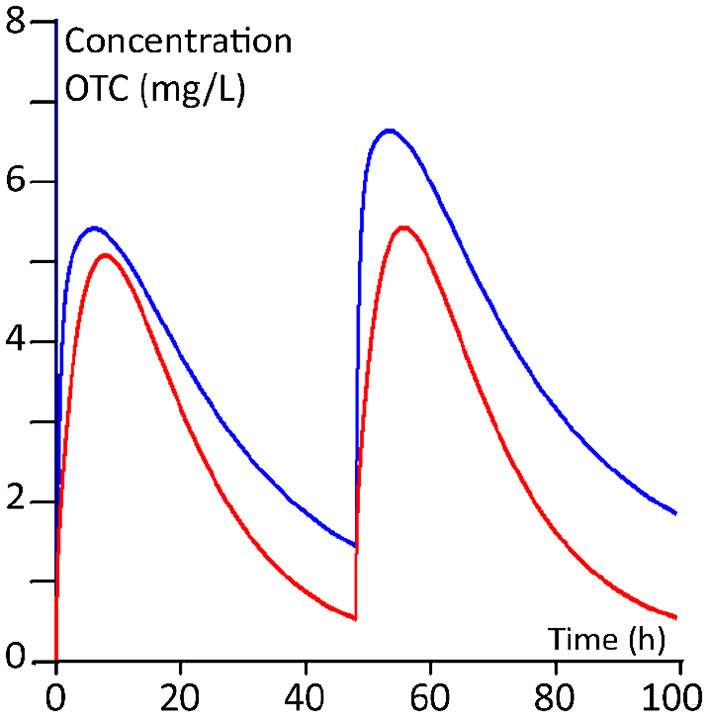
Twenty mg/kg regimen (twice with an interval of 2 days). Red line: 100 kg BW calves; blue line 400 kg BW calves.

The plasma kinetics, derived from the PBPK modelling of the two dosing regimens for 100 kg and 400 kg steers, are shown in Figures 5, 6.

The calculated median AUCs derived from the Monte Carlo simulations for days 1, 2, 3 and 4 for the daily 10 mg/kg regimen and for the twice 20 mg/kg regimen are given in Table 7. From the plasma concentration-time profiles as well as from the AUCs, it is clear that the younger calves of 100 kg have a lower OTC exposure compared to the older calves of 400 kg.

**Table 7:** Median AUCs at days 1, 2, 3 and 4 for the daily 10 mg/kg regimen and the twice 20 mg/kg regimen, for both 100 and 400 kg BW steer calves.

**Period**	**Dose (mg/kg)**	**AUC 400 kg BW calves (h.mg/L)**	**AUC 100 kg BW calves (h.mg/L)**
Day 1 (0–24)	10	54.7 ± 6.8	45.5 ± 7.7
Day 2 (24–48)	10	81.6 ± 9.6	61.7 ± 8.7
Day 3 (48–72)	10	94.0 ± 11.2	66.8 ± 9.5
Day 4 (72–96)	10	103.9 ± 13.2	68.6 ± 9.9
Day 1 (0–24)	20	108.9 ± 14,8	90.6 ± 14.2
Day 2 (24–48)	-	54.8 ± 8,7	33.3 ± 10.6
Day 3 (48–72)	20	136.3 ± 15.6	100.6 ± 14.1
Day 4 (72–96)	-	71.9 ± 13.6	36.8 ± 11.3

*Standard Deviations of the AUC's were derived using Monte Carlo simulations (5,000 runs) for each dosing regimen, bodyweight, and day, with random sampling from the distributions of the model parameters.

### PK/PD Analysis for the Two Commonly Used Dosing Regimens for Steers of Different Age

Based on multiple runs of the PBPK model (5,000 each), while randomly sampling from the distribution of the model parameters, the statistical significance of the differences in AUCs_24h_ for the two age groups were determined. Whereas the median AUC values for 100 kg calves were consistently lower, the difference (down to 52%) did not reach statistical significance. Further PBPK model calculations revealed that with the “long-acting” dosing regimen, the 100 kg calves would need two doses of 41 mg/kg, to reach an equal mean AUC on day 4 as the 400 kg calves given two doses of 20 mg/kg.

**Figure 7:**
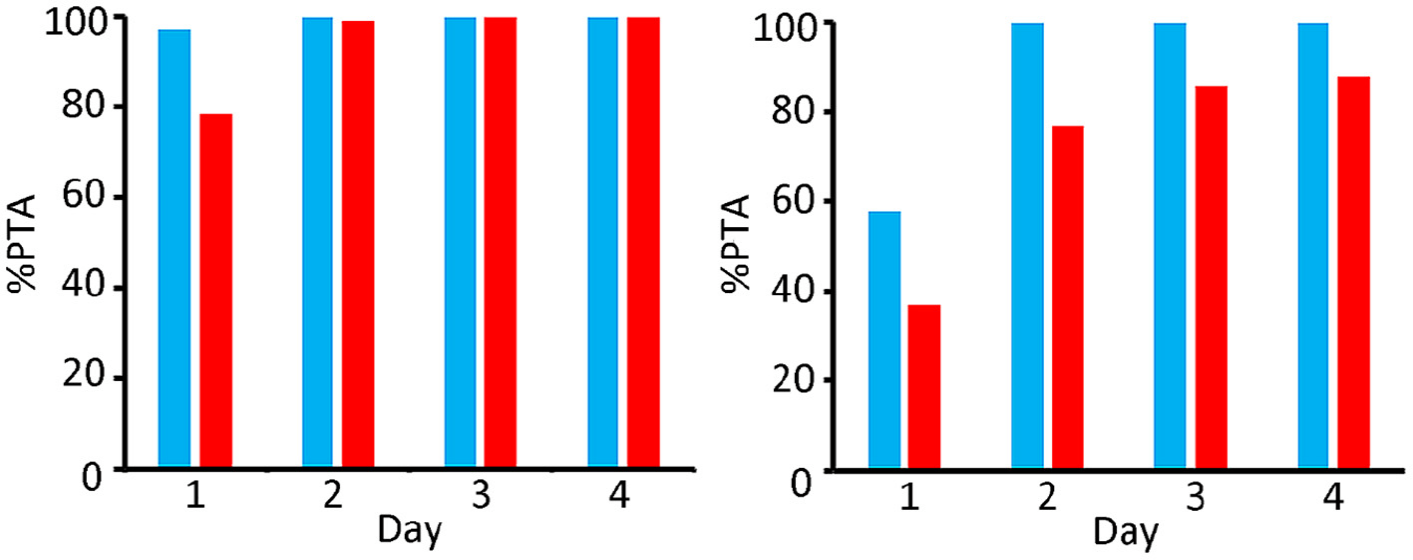
Comparison of PTA [%] for bacteriostatic effectivity (left) and bactericide effectivity (right) of OTC for *M. Haemolytica* in 400 kg (blue) and 100 kg (red) steers; dosing regimen 10 mg/kg per day during 4 consecutive day.

**Figure 8:**
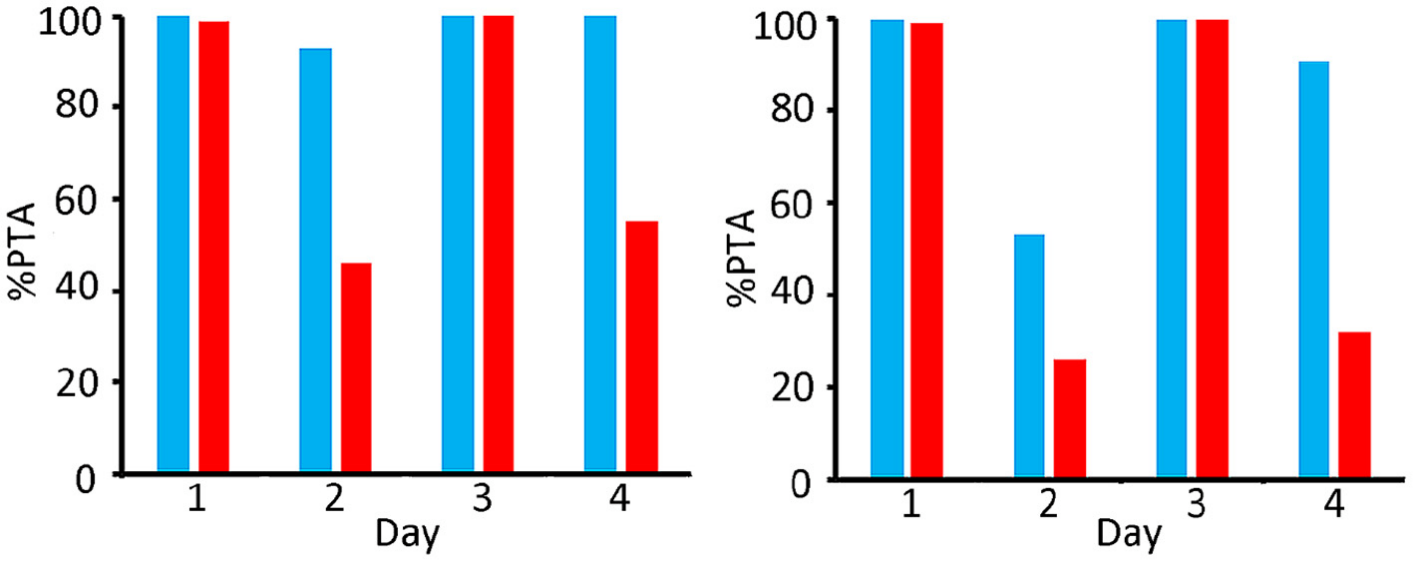
Comparison of PTA [%] for bacteriostatic effectivity (left) and bactericide effectivity (right) of OTC for *M. Haemolytica* in 400 kg (blue) and 100 kg (red) steers; dosing regimen 20 mg/kg twice (48 h dosing interval).

Monte Carlo simulations were made for both dosing regimens for 4 days, as described in 2.8, and the PTAs were calculated. Results for the 100 kg steers were compared to the results of the 400 kg steers (see Figures 7, 8).

When targeting a 90% PTA as an indication for efficacy [as proposed by (16)], Figure 7 shows that the short-acting (10 mg/kg) dosing regimen is effectively bacteriostatic for the whole 4 day period in the 400 kg group, and on days 2, 3, and 4 in the 100 kg group. As far as the bactericidal efficacy is concerned, this dosing regimen fulfils the criterium on days 2, 3 and 4 for the 400 kg group, whilst the targeted PTA for bactericidal efficacy was not reached at all over the entire 4 day period in the 100 kg group.

Figure 8 shows that, as far as the bacteriostatic efficacy is concerned, a dosing regimen of 20 mg/kg twice (48 h dosing interval) fulfils the PTA criterium over the entire period of 4 days in the 400 kg group, whilst this was the case only on days 1 and 3 for the 100 kg group. As far as the bactericidal efficacy is concerned, this dosing regimen fulfils the criterium on days 1, 3 and 4 for the 400 kg group, whilst this was only obtained at days 1 and 3 in the 100 kg group.

The long-acting regimen only fulfils the criterium of 90% PTA in the 100 kg group on days 1 and 3. The 400 kg group gives a better result with a sufficient PTA at days 1, 3 and 4.

The simulations indicate that for young calves of 100 kg a long-acting dosing regimen would be less effective, based on PK/PD considerations.

## Discussion

Several PBPK models have been constructed to describe the PK of OTC in various animal species, including cattle, sheep, humans, dogs, and salmon (13, 33–35). These models did not address the bodyweight-dependent PK of OTC, as described by Nouws et al. (36). To address this issue, our model includes a bodyweight-dependent renal clearance of the drug. This phenomenon is likely to be directly related to the glomerular filtration rate, which increases allometrically with bodyweight. To our knowledge, our PBPK model is the first to successfully incorporate the renal clearance scaled to the bodyweight of steer calves to adequately predict the PK of OTC in steers of different bodyweights. In addition, our model contains a bone-forming tissue compartment, which was not included in previous models for OTC. This compartment was introduced to address the high concentrations in bone and to allow for an adequate prediction of the prolonged terminal phase of the plasma concentrations.

It is generally indicated by the marketing authorisation holders of OTC products that the 100 mg/ml formulations with a 10 mg/kg bw dosing regimen are “short-acting formulations,” and that the 200 mg/ml formulations with a 20 mg/kg bw dosing regimen are “long-acting formulations.” It is remarkable that in our study, the fitted R_abs_ for both strengths were similar. Therefore, the difference in PK (Cmax) between the so-called short-acting (10 mg/kg bw) and long-acting (20 mg/kg bw) formulations appears to be determined by the difference in dose rather than by any specific formulation effects. Indeed, (37) reported that the differences between the composition of those formulations, in terms of inactive ingredients, are small.

Our PBPK model simulations showed that the PK in 100 kg animals was different from that of the 400 kg animals. This confirms the finding of Nouws et al. (36), who concluded, based on their experimental data, that young calves may require a double dose in order to achieve the same plasma levels as in adult animals. Our simulations showed a difference in AUCs, although not statistically significant, and indeed a double dose would be needed for 100 kg calves to achieve the same mean AUCs_24h_ as the 400 kg calves on all four days. This is of particular importance, because for OTC the AUC_24h_/MIC is the relevant PK/PD index, and therefore a difference in AUC may negatively impact efficacy. Our PK/PD analysis, conducted for 100 kg and 400 kg animals separately, indicated that the PTA for *M. haemolytica* was <90% in calves of 100 kg, in particular on the second and fourth day of the “long-acting” dosing regimen of 2 x 20 mg/kg with an interval of 48 h, which is used for the treatment of BRD in cattle. However, the differences in AUCs_24h_ did not appear to meaningfully impact on the PTA in simulations of the 10 mg/kg once a day “short-acting” dosing regimen.

The outcome of these “*in silico*” experiments suggests that the age and weight of the animals should be considered as a factor in the choice for a dosing regimen for the treatment of BRD in steer calves.

PBPK modelling is a useful tool to identify the variation in PK, related to differences in physiological parameters in different subpopulations of the target animals. The availability of validated PBPK models may reduce the need for *in vivo* studies. In our simulations, we showed the applicability of PBPK modelling considering age-dependent renal clearance of OTC. However, PBPK modelling could also be used to show PK variation according to for example breed, production stage, health status, or age, as long as the underlying mechanisms of that variation are known. PBPK modelling can be used to identify specific subpopulations that may require further consideration of the effective dose and/or the withdrawal period.

## Conclusion

The integration of PBPK and PD for OTC used for the treatment of BRD in calves indicated that the PTA for *M. haemolytica*, the dose-limiting pathogen, was sufficient for efficacy in calves of 400 kg, but insufficient for calves of 100 kg, when using a long acting dosing regimen of 20 mg/kg BW, twice, with a 48-h interval. The use of another dosing regimen of 10 mg/kg BW/day for 4 days predicted sufficient PTAs in both age groups.

## Data Availability Statement

The original contributions presented in the study are included in the article/supplementary material, further inquiries can be directed to the corresponding author.

## Author Contributions

PH, JS, and RG conceptualised the model. PH implemented the model and performed the simulations. JS drafted the introduction and discussion. RG contributed to the discussion and provided a clinical perspective to the interpretation. All authors contributed to the interpretation of the results and approved the final manuscript.

## Funding

Open access publication of this study is made possible in part by institutional support by Utrecht University.

## Conflict of Interest

The authors declare that the research was conducted in the absence of any commercial or financial relationships that could be construed as a potential conflict of interest. The handling editor JM declared shared consortiums with one of the authors RG, at time of review.

## Publisher's Note

All claims expressed in this article are solely those of the authors and do not necessarily represent those of their affiliated organizations, or those of the publisher, the editors and the reviewers. Any product that may be evaluated in this article, or claim that may be made by its manufacturer, is not guaranteed or endorsed by the publisher.

## Footnotes

^1^In order to calculate the concentration in a compartment both sides of the mass balance equation have to be divided by weight (fVol.BW) for that compartment.

## References

[1] Mzyk DA Bublitz CM Martinez MN Davis JL Baynes RE Smith GW. Impact of bovine respiratory disease on the pharmacokinetics of danofloxacin and tulathromycin in different ages of calves. PLoS ONE. (2019) 14:e0218864. 10.1371/journal.pone.0218864 31233558 PMC6590872

[2] Igarza L Soraci A Auza N Zeballos H. Some pharmacokinetic parameters of R-(–)-and S-(+)-ketoprofen: the influence of age and differing physiological status in dairy cattle. Vet Res Commun. (2004) 28:81–7. 10.1023/B:VERC.0000009534.64533.b9 14989364

[3] Gorden PJ Burchard M Ydstie JA Kleinhenz MD Wulf LW Rajewski SJ. Comparison of milk and plasma pharmacokinetics of meloxicam in postpartum versus mid-lactation Holstein cows. J Vet Pharmacol Ther. (2018) 41:463–8. 10.1111/jvp.12488 29430684

[4] Warner R Ydstie JA Wulf LW Gehring R Coetzee JF Mochel JP. Comparative pharmacokinetics of meloxicam between healthy post-partum vs. mid-lactation dairy cattle. Front Vet Sci. (2020) 7:548. 10.3389/fvets.2020.00548 33102542 PMC7506135

[5] Chang SK Davis JL Cheng CN Shien RH Hsieh MK Koh BW. Pharmacokinetics and tissue depletion of florfenicol in Leghorn and Taiwan Native chickens. J Vet Pharmacol Ther. (2010) 33:471–9. 10.1111/j.1365-2885.2009.01155.x 20840391

[6] Howard JT Baynes RE Brooks JD Yeatts JL Bellis B Ashwell MS. The effect of breed and sex on sulfamethazine, enrofloxacin, fenbendazole and flunixin meglumine pharmacokinetic parameters in swine. J Vet Pharmacol Ther. (2014) 37:531–41. 10.1111/jvp.12128 24731191

[7] Nouws JFM Vree TB Termond E Lohuis J Van Lith P Binkhorst GJ. Pharmacokinetics and renal clearance of oxytetracycline after intravenous and intramuscular administration to dairy cows. Vet Q. (1985) 7:296–305. 10.1080/01652176.1985.9694003 4071950

[8] De Laistre Banting A. Subcutaneous and intramuscular injection of oxytetracycline in calves: comparison of serum concentration and local tolerance. J Vet Pharmacol Ther. (1987) 10:184–6. 10.1111/j.1365-2885.1987.tb00099.x 3612949

[9] Meijer LA Ceyssens KGF De Jong WT Greve BD. Three phase elimination of oxytetracycline in veal calves; the presence of an extended terminal elimination phase. J Vet Pharmacol Ther. (1993) 16:214–22. 10.1111/j.1365-2885.1993.tb00166.x 8345571

[10] Nouws JFM Vree TB. Effect of injection site on the bioavailability of an oxytetracycline formulation in ruminant calves. Vet Q. (1983) 5:165–70. 10.1080/01652176.1983.9693891 6649399

[11] Mevius DJ Nouws JFM Breukink HJ Vree TB Driessens F Verkaik R. Comparative pharmacokinetics, bioavailability and renal clearance of five parenteral oxytetracycline-20% formulations in dairy cows. Vet Q. (1986) 8:285–94. 10.1080/01652176.1986.9694057 3798710

[12] Terhune TN Upson DW. Oxytetracycline pharmacokinetics, tissue depletion, and toxicity after administration of a long-acting preparation at double the label dosage. J Am Vet Med Assoc. (1989) 194:911–7. 2703423

[13] Achenbach TE. Physiological and classical pharmacokinetic models of oxytetracycline in cattle (thesis). Simon Fraser University, Burnaby, BC, Canada (2000).

[14] Clarke CR Wang Z Cudd L Burrows GE Kirkpatrick JG Brown MD. Pharmacokinetics of two long-acting oxytetracycline products administered subcutaneously and intramuscularly. J Vet Pharmacol Ther. (1999) 22:65–7. 10.1046/j.1365-2885.1999.00181.x 10211720

[15] Toutain PL Raynaud JP. Pharmacokinetics of oxytetracycline in young cattle: comparison of conventional vs long-acting formulations. Am J Vet Res. (1983) 44:1203–9. 6881660

[16] Lees P Potter T Pelligand L Toutain PL. Pharmacokinetic–pharmacodynamic integration and modelling of oxytetracycline for the calf pathogens Mannheimia haemolytica and Pasteurella multocida. J Vet Pharmacol Ther. (2018) 41:28–38. 10.1111/jvp.12439 28736817

[17] Breeze R Gay C. Plasma levels of a long-acting oxytetracycline in cattle. Bovine Pract. (1981) 16:22–3.1491343

[18] Davey LA Ferber MT Kaye B. Comparison of the serum pharmacokinetics of a long acting and a conventional oxytetracycline injection. Vet Record. (1985) 117:426–9. 10.1136/vr.117.17.426 4071932

[19] Craigmill AL Holland RE Robinson D Wetzlich S Arndt T. Serum pharmacokinetics of oxytetracycline in sheep and calves and tissue residues in sheep following a single intramuscular injection of a long-acting preparation. J Vet Pharmacol Ther. (2000) 23:345–52. 10.1046/j.1365-2885.2000.00292.x 11168911

[20] Lin Z Li M Wang YS Tell LA Baynes RE Davis JL. Physiological parameter values for physiologically based pharmacokinetic models in food-producing animals. Part I: Cattle and swine. J Vet Pharmacol Ther. (2020) 43:385–420. 10.1111/jvp.12861 32270548 PMC7540321

[21] Lautz LS. Towards next generation risk assessment of chemicals: development and application of physiologically based kinetic models in farm animals (Ph.D. dissertation). Nijmegen: Radboud University (2020).

[22] Murayama I Miyano A Sasaki Y Kimura A Sato S Furuhama K. Glomerular filtration rate in Holstein dairy cows estimated from a single blood sample using iodixanol. J Dairy Sci. (2013) 96:5120–8. 10.3168/jds.2013-6884 23791486

[23] Yamaoka K Nakagawa T. A nonlinear least squares program based on differential equations, MULTI (RUNGE), for microcomputers. J Pharmacobio Dyn. (1983) 6:595–606. 10.1248/bpb1978.6.595 6689031

[24] Li M Gehring R Riviere JE Lin Z. Development and application of a population physiologically based pharmacokinetic model for penicillin G in swine and cattle for food safety assessment. Food Chem Toxicol. (2017) 107:74–87. 10.1016/j.fct.2017.06.023 28627373

[25] Landoni MF Errecalde JO. Tissue concentrations of a long-acting oxytetracycline formulation after intramuscular administration in cattle. Rev Sci Tech. (1992) 11:909–909. 10.20506/rst.11.3.635 1472735

[26] Cornejo J Pokrant E Araya D Briceño C Hidalgo H Maddaleno A. Residue depletion of oxytetracycline (OTC) and 4-epi-oxytetracycline (4-epi-OTC) in broiler chicken's claws by liquid chromatography-tandem mass spectrometry (LC-MS/MS). Food Addit Contam Part A. (2017) 34:494–500. 10.1080/19440049.2016.1263876 27879173

[27] Korner U Kuhne M Wenzel S. Tetracycline residues in meat and bone meals. Part 1: Methodology and examination of field samples. Food Addit Contam. (2001) 18:293–302. 10.1080/02652030121556 11339263

[28] Varela Cruz NP. Slaughtered hogs with discoloured bones and the relationship with tetracycline medication in the grower-finisher stage. University of Guelph, Guelph, ON, Canada (2012).

[29] Marenzana M Arnett TR. The key role of the blood supply to bone. Bone Res. (2013) 1:203–15. 10.4248/BR201303001 26273504 PMC4472103

[30] Brentnall C Cheng Z McKellar QA Lees P. Pharmacokinetic–pharmacodynamic integration and modelling of oxytetracycline administered alone and in combination with carprofen in calves. Res Vet Sci. (2013) 94:687–94. 10.1016/j.rvsc.2013.01.012 23415880

[31] de Jong A Thomas V Simjee S Moyaert H El Garch F Maher K. Antimicrobial susceptibility monitoring of respiratory tract pathogens isolated from diseased cattle and pigs across Europe: the VetPath study. Vet Microbiol. (2014) 172:202–15. 10.1016/j.vetmic.2014.04.008 24837878

[32] El Garch F de Jong A Simjee S Moyaert H Klein U Ludwig C. Monitoring of antimicrobial susceptibility of respiratory tract pathogens isolated from diseased cattle and pigs across Europe, 2009–2012: VetPath results. Vet Microbiol. (2016) 194:11–22. 10.1016/j.vetmic.2016.04.009 27102206

[33] Craigmill AL. A physiologically based pharmacokinetic model for oxytetracycline residues in sheep. J Vet Pharmacol Ther 26. (2003) 55–63. 10.1046/j.1365-2885.2003.00451.x 12603776

[34] Lin Z Li M Gehring R Riviere JE. Development and application of a multiroute physiologically based pharmacokinetic model for oxytetracycline in dogs and humans. J Pharm Sci. (2015) 104:233–43. 10.1002/jps.24244 25407474

[35] Law FCP. A physiologically based pharmacokinetic model for predicting the withdrawal period of oxytetracycline in cultured Chinook salmon *(Oncorhynchus tshawytscha*). In: Smith DJ Gingerich WH Beconi-Barker MG, editors. Xenobiotics in Fish. Boston, MA: Springer (1999). p. 105–21. 10.1007/978-1-4615-4703-7_8

[36] Nouws JFM Van Ginneken CAM Ziv G. Age-dependent pharmacokinetics of oxytetracycline in ruminants. J Vet Pharmacol Ther. (1983) 6:59–66. 10.1111/j.1365-2885.1983.tb00455.x 6854733

[37] CVMP. Reflection paper on dose review and adjustment of established veterinary antibiotics in the context of SPC harmonization. Eur Med Agency. EMA/CVMP/849775/2017[1]:1–137. (2021).

